# Conventional suture with prolonged timing of drainage is as good as quilting suture in preventing seroma formation at pectoral area after mastectomy

**DOI:** 10.1186/s12957-021-02257-8

**Published:** 2021-05-12

**Authors:** Juan Huang, Shouman Wang, Yuhui Wu, Jian Hai, Jie Mao, Xue Dong, Zhi Xiao

**Affiliations:** 1 Breast Cancer Center, Department of General Surgery, Xiangya Hospital, Central South University, 87 Xiangya Road, Changsha, Hunan People’s Republic of China 410008; 2 Clinical Research Center For Breast Cancer Control and Prevention In Hunan Province, Changsha, China

**Keywords:** Quilting suture, Drainage time, Seroma, Mastectomy

## Abstract

**Background:**

The aim of this study was to compare conventional suture with prolonged timing of drainage with quilting suture on the formation of seroma at pectoral area after mastectomy (ME) with sentinel lymph node biopsy (SLN) or axillary lymph node dissection (ALND) for breast cancer.

**Methods:**

Three hundred and eighty-eight consecutive breast cancer patients were retrospectively analyzed and categorized into three groups. Patients in group 1 were with quilting suture, group 2 with conventional suture and 13–15 days drainage in situ, and group 3 with conventional suture and 20–22 days drainage. The primary outcome was the incidence of grades 2 and 3 seroma at anterior pectoral area within 1 month postoperatively. Cox regression was used for analysis.

**Results:**

The incidence of grades 2 and 3 seroma was comparable among groups (9.5% vs. 7.9% vs. 5.3%, *p* = 0.437), as well as late grades 2 and 3 seroma among groups (4.3% vs. 2.9% vs. 1.5%, *p* = 0.412). Old age, high body mass index, and hypertension were independent risk factors for grades 2 and 3 seroma.

**Conclusions:**

Prolonged timing of drainage to 13–15 days in conventional suture was long enough to decrease the incidence of grades 2 and 3 seroma as lower as that in quilting suture group at pectoral area within 1 month after mastectomy.

## Background

The incidence of breast cancer is increasing year by year in China, and mastectomy with SLN or ALND dissection is the most popular surgical treatment for it [1]. The seroma in axilla and dead space beneath skin flaps after surgery would lead to discomfort, repeat visit to outpatient clinics, and surgical site infection [2, 3]. Many clinical studies have tried various methods to lower seroma formation, such as suction drainage, shoulder immobilization, quilting sutures, axillary exclusion, fibrin sealants, and thrombin sealants [4–11].

In our previous retrospective study, we focus on the seroma in the dead space at the pectoral area [12]. We showed that quilting suture applied at medial and inferior border of the dead space at the pectoral area could reduce the incidence of grades 2 and 3 seroma compared with the conventional suture. Furthermore, we found that quilting suture could significantly lower the incidence of late grades 2 and 3 seroma which occurred after the removal of drainage system, rather than the early grades 2 and 3 seroma. In order to lower the incidence of late grades 2 and 3 seroma, we tried to prolong the time of drainage and explore the best time point of drainage removal in this study.

## Methods

### Patients

We retrospectively analyzed 388 consecutive breast cancer patients who underwent ME plus SLN or ALND between September 2017 and August 2020 in the Breast Surgery Department of Xiangya Hospital, Central South University, Changsha City, P.R. China. Patients treated with bilateral breast mastectomy surgery, breast-conserving surgery, breast reconstruction surgery, and skin grafting, and patients with scleroderma or systemic lupus erythematosus were excluded. All patients were female and local citizens older than 18 years and were first diagnosed as breast invasive carcinoma or ductal carcinoma in situ. Patients were divided into 3 groups. During September 2017 to January 2019, 116 patients in group 1 with quilting suture were included for analysis, and these patients were also enrolled in our previous study [12]. The drainage system was removed 5–9 days postoperatively in these patients with quilting technique. During February 2019 and October 2019, 139 patients in group 2 with conventional suture and drainage system removal around 13–15 days postoperatively were included in this study. During November 2019 and August 2020, 133 patients in group 3 with conventional suture and drainage system removal around 20–22 days postoperatively were included in this study. Patients’ data such as age, BMI, medical history, familial history of cancer, TNM stage, and surgical method were collected from electronic medical record. This study was reviewed by the Institutional Ethical Review Board of Xiangya Hospital.

### Surgical technique

The procedure of surgical technique was described in our previous study [12]. All the patients had a closed suction drain in the axillary area. Patients with quilting suture were in group 1, and the skin flaps were sutured to the underlying pectoralis major with 10–12 stitches in one row 1–2 cm away from the medial and inferior border of the dead space which were the most common places of seroma formation, without a drainage for this area. The drainage in the axilla was removed around 5–9 days postoperatively. For conventional wound suture in groups 2 and 3, the skin flaps were not fixed subcutaneously. There were two drainages, one for axilla and one for the dead space at the pectoral area. The second drain was inserted beneath the flaps near the medial and inferior border of the dead space at the pectoral area. The two drainages were removed 13–15 days in group 2 or 20–22 days in group 3 postoperatively.

All patients were discharged 5–9 days postoperatively after they had received the first cycle of chemotherapy if needed. The follow-up visit was recommended at least twice within 1 month after operation or 1 week after removal of drainage, and physical and/or ultrasound examination were recommended during the visit.

### Outcomes

The primary outcome in this study was the incidence of grade 2 and grade 3 seroma in the pectoral area within 1 month postoperatively. We applied the criteria for adverse events classification (CTCAE) 4.0 to categorize the seroma into grade 1, grade 2, and grade 3 which were described as that in our previous study. In short, grade 1 seroma is asymptomatic and intervention is not indicated; grades 2 and 3 are symptomatic and interventions are indicated. The secondary outcomes were the overall (grade 1, 2, or 3) seroma rate in the pectoral area, rate of wound hematoma indicated with intervention of puncture or surgery, surgical site infection, inadequate wound healing, hospital stay after surgery (days), and drainage volume (milliliters). We categorized seroma into early or late seroma according to the drainage removal time. Early seroma was defined as seroma formed before removal of drainage, and late seroma was defined as seroma formed after removal of drainage at any time but within 1 month postoperatively. Only the seroma at the pectoral area was recorded in this study.

### Influence-on-life assessment

Patients with grades 2 and 3 seroma treated with aspiration were questioned to assess the influence of drainage in situ on quality of life. There were two questions. One was whether the drainage in situ had a great impact on life; another question was whether patients would like to prolong the time of drainage in situ to avoid or decrease the incidence of grades 2 and 3 seroma or repeat visit to outpatient clinics.

### Statistical analysis

The characteristics of patients were compared among groups 1, 2, and 3. Categorical variables were analyzed by *χ*^2^ test or Fisher exact test, and continuous variables were analyzed by *one-way ANOVA* test. Statistical significance was defined at *p* value < 0.05. SPSS version 19.0 was used for statistical analysis.

## Results

### Patient characteristics

The study enrolled 116 patients in group 1 with quilting suture but without drainage of pectoral area and 139 patients in group 2 with drainage of pectoral area which was removed 13–15 days postoperatively and 133 patients in group 3 with drainage removal 20–22 days postoperatively. The patient characteristics were similar among these three groups (Table 1).

**Table 1:** Patient characteristics

		Quilting suture (group 2) *n*=116	Conventional suture (group 2) *n*=139	Conventional suture (group 3) *n*=133	*p* value
Age (years)	Mean (SD)	52.7 (9.9)	52.3 (9.3)	51.6 (11.3)	0.780
BMI (kg/m^2^)	Mean (SD)	22.7 (2.9)	23.6 (3.1)	22.9 (3.3)	0.197
Hypertension	*n* (%)	20 (17.2)	21 (15.1)	19 (14.8)	0.435
Diabetes	*n* (%)	4 (3.4)	5 (3.6)	2 (1.5)	0.520
Smoking	*n* (%)	3 (2.6)	2 (1.4)	1 (0.8)	0.500
Menopause	*n* (%)	69 (59.5)	77 (55.4)	63 (47.4)	0.145
Familial history of cancer	*n* (%)	24 (20.7)	20 (14.4)	25 (18.8)	0.395
Neoadjuvant chemotherapy	*n* (%)	23 (19.8)	30 (21.6)	22 (16.5)	0.567
Surgery					
ME+SLN	*n* (%)	18 (15.5)	32 (23.0)	36 (27.1)	0.087
ME+ALND	*n* (%)	98 (84.5)	107 (77.0)	97 (72.9)	
TNM stage					
I	*n* (%)	37 (31.9)	33 (23.7)	43 (32.3)	0.121
II	*n* (%)	67 (57.8)	90 (64.7)	67 (50.4)	
III	*n* (%)	12 (10.3)	16 (11.6)	23 (17.3)	

Discrete variables used *χ*^2^ test or Fisher exact test; continuous variables *one-way ANOVA* test. The patient characteristics were similar among the three groups

*BMI* body mass index (calculated as weight in kilograms divided by height in meters squared), *ME* mastectomy, *SLN* sentinel lymph node biopsy, *ALND* axillary lymph node dissection, *SD* standard deviation

### Outcome comparisons

The incidence of grades 2 and 3 seroma within 1 month after operation was similar in group 1, group 2, and group 3 (9.5% vs. 7.9% vs. 5.3%, *p* = 0.437). The rates of early or late seroma in grades 2 and 3 were not significantly different among the three groups (*p* =0.837 or 0.412, respectively) as shown in Table 2. The incidence of grade 1 seroma was comparable among the three groups (*p* = 0.780).

**Table 2:** Binary outcome comparisons

		Quilting suture (group 2) *n*=116	Conventional suture (group 2) *n*=139	Conventional suture (group 3) *n*=133	*p* value
Seroma (grade 1, early)	*n* (%)	14 (12.1)	12 (8.6)	12 (9.0)	0.612
Seroma (grades 2–3, early)	*n* (%)	6 (5.2)	7 (5.0)	5 (3.8)	0.837
Seroma (grade 1, late)	*n* (%)	7 (6.0)	9 (6.5)	6 (4.5)	0.767
Seroma (grades 2–3, late)	*n* (%)	5 (4.3)	4 (2.9)	2 (1.5)	0.412
Seroma (grade 1)	*n* (%)	21 (18.1)	21 (15.1)	18 (13.5)	0.780
Seroma (grades 2–3)	*n* (%)	11 (9.5)	11 (7.9)	7 (5.3)	0.437
Seroma	*n* (%)	32 (27.6)	32 (23.0)	25 (18.8)	0.258
Hematoma	*n* (%)	5 (4.3)	5 (3.6)	3 (2.3)	0.654
Surgical site infection	*n* (%)	4 (3.4)	2 (1.4)	2 (1.5)	0.251
Inadequate wound healing	*n* (%)	2 (1.7)	2 (1.4)	3 (2.3)	0.580

Discrete variables used χ^2^ test or Fisher exact test. Outcome comparisons among the three groups. The incidence of grades 2 and 3 seroma or late seroma in grades 2 and 3 was similar among the three groups (*p* = 0.437 and *p* = 0.412 respectively). The incidence of grade 1 seroma was comparable among the three groups (*p* = 0.780)

There were no significant differences among the three groups regarding hematoma, surgical site infection, inadequate wound healing, and length of hospital stay after operation (Tables 2 and 3). Patients treated with quilting suture were more likely to experience less volume of drainage than those in group 2 and group 3 with conventional suture (374.9 vs. 439.1 vs. 461.4 ml, *p* <0.001) as shown in Table 3.

**Table 3:** Continuous outcome comparisons

		Quilting suture (group 2) *n*=116	Conventional suture (group 2) *n*=139	Conventional suture (group 3) *n*=133	*p* value
Hospital stay after surgery (days)	Mean (SD)	7.8 (1.0)	7.9 (1.0)	7.7 (1.1)	0.609
Drain volume (ml)	Mean (SD)	374.9 (57.1)	439.1 (66.9)	461.4 (82.3)	<0.001

Continuous variables used *one-way ANOVA* test. Outcome comparisons among the three groups. Quilting suture was significantly associated with less volume of drainage compared with that of conventional suture groups 2 and 3 (*p* < 0.001)

*SD* standard deviation

### Risk factors

A multivariate logistic regression was used to evaluate the risk factors of grades 2 and 3 seroma formation as shown in Table 4. Old age, high BMI, and hypertension were found to be risk factors for grades 2 and 3 seroma. Patients younger than 60-year-old or with BMI less than 25 or without hypertension experienced much less grades 2 and 3 seroma within 1 month postoperatively when compared to patients older than 60 or with BMI higher than 25 or with hypertension (*p* < 0.05). Diabetes, neoadjuvant chemotherapy, and type of suture had no influence on developing grades 2 and 3 seroma in the pectoral area.

**Table 4:** Risk factors for seroma (grades 2–3)

Parameter	Seroma (grades 2–3)	
	OR (95% CI)	*p* value
Age (year) (≥ 60 or <60)	3.020–19.811	<0.001
BMI (kg/m^2^) (≥25 or <25)	5.422–38.972	<0.001
Hypertension	1.346–10.208	0.011
Diabetes	0.061–4.295	0.537
Neoadjuvant chemotherapy	0.142–2.317	0.436
Suture type		
Group 2 vs group 1	0.200–1.658	0.307
Group 3 vs group 1	0.138–1.397	0.164

*ME* mastectomy, *BMI* body mass index

A multivariate logistic regression of the risk factors of seroma (grades 2–3). Old age, high BMI, and hypertension were independent risk factors for early and late seroma in grades 2–3 (*p* < 0.05). Different suture types with various duration of drainage system did not influence grades 2 and 3 seroma formation

### Influence-on-life assessment

A total of 18 patients in groups 2 and 3 with grades 2 and 3 seroma were assessed. Three (16.7%) patients had the feeling that drainage in situ had a great impact on their life. One (5.6%) patient would like to choose aspiration rather than prolong the time of drainage in situ. Most patients had acceptable discomfort (83.7%) and would like to prolong the time of drainage in situ to avoid or decrease the incidence of grades 2 and 3 seroma (94.4%) as shown in Table 5.

**Table 5:** Quality of life questionnaire survey

	Yes	No
Great impact on life of drainage	3 (16.7)	15 (83.3)
Willing of extension of drainage time	17 (94.4)	1 (5.6)

A total of 18 patients in groups 2 and 3 with grades 2 and 3 seroma were assessed

## Discussion

This study showed that the same effect could be achieved as that of quilting suture by prolonging the time of drainage in situ around 2 weeks postoperatively in conventional suture group concerning to the formation of seroma in grades 2 and 3 within 1 month after mastectomy with SLN or ALND. This study also showed that there was no need to prolong the drainage removal time more than 2 weeks; old age, high BMI, and hypertension were risk factors on postoperative seroma formation, consistent with previous results from similar studies [6, 13–16].

In our previous study, we showed that conventional suture led to a high incidence of late grades 2 and 3 seroma forming after drainage removal [12]. We tried to find a way to decrease grades 2 and 3 seroma in the conventional suture group. We prolonged the duration of drainage in situ, from the day of removal around 5–9 days to 2 or 3 weeks postoperatively. In our previous study, the timing of drainage removal was 5–9 days in both conventional and quilting suture groups. The incidences of late grades 2 and 3 seroma were 15.1% in conventional group and 4.3% in quilting group. In this study, we removed the drainage on 13–15 days postoperatively in conventional suture group 2, and on 20–22 days in group 3. The results showed that the incidence of late grades 2 and 3 seroma was as low as that in quilting suture group, without significant difference (*p* =0.412). This meant that extension of drainage in situ could decrease the formation of grades 2 and 3 seroma.

There were some controversies about the time of drain removal. Some studies showed that seroma was not reduced by prolonged suction drainage of the wound, and short-time drainage of 3–6 days was safe, economical, and acceptable [17, 18]. Some others showed that early removal of drainage was not beneficial and tried to find complementary solution for it [19–21]. In this study, the data showed that the removal of drainage on 13–15 days postoperatively was the suitable time point and was long enough to reduce the incidence of grades 2 and 3 seroma. There was no need to prolong the drainage in situ to 20–22 days which only achieved a 1.4% reduction of incidence of late grades 2 and 3 seroma. A study from Gupta et al. showed that 8-day drainage had significant advantages in reducing the number of aspiration and total aspiration volume compared to 5-day drainage, and the number of lymphoceles drained in the 5-day group was significantly higher than the 8-day group (48% vs. 28%) [22]. The incidence of 28% in the 8-day group was comparable with the results in our previous study with an incidence of 19.3% of grades 2 and 3 seroma in the conventional group with drainage in situ 5–9 days (mean 7.8 days). Although the study from Gupta et al. did not categorize the seroma according to the severity, there were a total of 42 aspirations for the lymphoceles of 16 (28%) patients. This implied that these aspirated lymphoceles might be grades 2 and 3 according the criteria for adverse events classification (CTCAE) 4.0. In our opinion, the incidence 19.3% of grades 2 and 3 seroma was quite high, and aspirations of seroma were not of economical benefit. This study showed that the extension of 5–7 more days of drainage was a solution to this problem, and what more was that extension to 20–22 days postoperatively was unnecessary and superfluous.

Some studies implied that long drainage time might be associated with higher surgical site infection and discomfort [19, 23–27]. In fact, not only in this study but also in our previous study, there was no significant difference of surgical site infection among these groups with various duration of drainage. In our opinion, we could avoid the surgical site infection by obeying aseptic principle during the duration of drainage in situ. Furthermore, some meta-analyses showed that more drainages for the seroma did not increase the surgical site infection [28, 29]. Concerning to the drainage discomfort of patients, only 16.7% of patients in this study thought that drainage had a great impact on their daily life, and 83.3% patients experienced minor discomfort and got used to the drainage system. Compared to the repeated visit to outpatient clinics, most patients (94.4%) would like to prolong the duration of drainage in situ which would not bring too much trouble to their daily life. Another reason for this was that repeated visit to outpatient clinics was costly and time-consuming. The only patient who preferred repeated visit to outpatient clinics and aspiration was a 46-year-old lady and wanted to return to work without a drainage system. This was quite different from the results of Stanczyk et al. which showed that most of patients (81%) preferred for early drain removal, even with a high incidence of seroma (84%) [18]. For all the patients with drainage system, it was their urgent desire to remove the drainage as soon as possible. But for those patients who had experienced symptomatic seroma after the removal of drainage system and visit outpatient repeatedly, they would be likely to change their original desire to keep the drainage system for longer time, as shown in our study. In a word, extension of the duration of drainage in situ was safe and acceptable.

There are some limitations in this study. First, this was a retrospective study without randomization. There were several clinical trials that have been processed to evaluate the effect of flap fixation on the formation of seroma [30, 31]. More useful information would be obtained from these studies in the near future. Second, the influence-on-life assessment was simple and subjective. Homogenized questionnaire is needed to better evaluate the impact of drainage on daily life. Third, we only assessed the impact of drainage on patients with grades 2 and 3 seroma in groups 2 and 3. Evaluation of all the patients in this study would be more convincing.

## Conclusion

Prolonged timing of drainage postoperatively in patients with conventional suture could reduce the incidence of grades 2 and 3 seroma as lower as that in quilting suture group at pectoral area within 1 month after mastectomy. The suitable time point of drainage removal was 13–15 days postoperatively. There was no need for extension of drainage removal to 20–22 days postoperatively.

## Data Availability

The datasets generated and/or analyzed during the current study are not publicly available due to its usage for other article, but are available from the corresponding author on reasonable request.

## Abbreviations

ME: Mastectomy
SLN: Sentinel lymph nodes biopsy
ALND: Axillary lymph nodes dissection
BMI: Body mass index

## References

[1] Li J, Zhang BN, Fan JH, Pang Y, Zhang P, Wang SL, Zheng S, Zhang B, Yang HJ, Xie XM (2011). A nation-wide multicenter 10-year (1999-2008) retrospective clinical epidemiological study of female breast cancer in China. BMC cancer.

[2] Ebner F, Friedl TWP, de Gregorio A, Lato K, Bekes I, Janni W, de Gregorio N (2018). Seroma in breast surgery: all the surgeons fault?. Arch Gynecol Obstet.

[3] Hashemi E, Kaviani A, Najafi M, Ebrahimi M, Hooshmand H, Montazeri A (2004). Seroma formation after surgery for breast cancer. World J Surg Oncol.

[4] Turner EJ, Benson JR, Winters ZE (2014). Techniques in the prevention and management of seromas after breast surgery. Future Oncol.

[5] Carless PA, Henry DA (2006). Systematic review and meta-analysis of the use of fibrin sealant to prevent seroma formation after breast cancer surgery. Br J Surg.

[6] Kuroi K, Shimozuma K, Taguchi T, Imai H, Yamashiro H, Ohsumi S, Saito S (2006). Evidence-based risk factors for seroma formation in breast surgery. Japanese J Clin Oncol.

[7] Conversano A, Mazouni C, Thomin A, Gaudin A, Fournier M, Rimareix F, Bonastre J (2017). Use of low-thrombin fibrin sealant glue after axillary lymphadenectomy for breast cancer to reduce hospital length and seroma. Clin Breast Cancer.

[8] Mortenson MM, Xing Y, Weaver S, Lee JE, Gershenwald JE, Lucci A, Mansfield PF, Ross MI, Cormier JN (2008). Fibrin sealant does not decrease seroma output or time to drain removal following inguino-femoral lymph node dissection in melanoma patients: a randomized controlled trial (NCT00506311). World J Surg Oncol.

[9] Faisal M, Abu-Elela ST, Mostafa W, Antar O (2016). Efficacy of axillary exclusion on seroma formation after modified radical mastectomy. World J Surg Oncol.

[10] van Bastelaar J, Beckers A, Snoeijs M, Beets G, Vissers Y (2016). Flap fixation reduces seroma in patients undergoing mastectomy: a significant implication for clinical practice. World J Surg Oncol.

[11] Sakkary MA (2012). The value of mastectomy flap fixation in reducing fluid drainage and seroma formation in breast cancer patients. World J Surg Oncol.

[12] Wu Y, Wang S, Hai J, Mao J, Dong X, Xiao Z (2020). Quilting suture is better than conventional suture with drain in preventing seroma formation at pectoral area after mastectomy. BMC Surg.

[13] ten Wolde B, van den Wildenberg FJ, Keemers-Gels ME, Polat F, Strobbe LJ (2014). Quilting prevents seroma formation following breast cancer surgery: closing the dead space by quilting prevents seroma following axillary lymph node dissection and mastectomy. Ann Surg Oncol.

[14] Oliveira MMF, Gurgel MSC, Amorim BJ, Ramos CD, Derchain S, Furlan-Santos N, Dos Santos CC, Sarian LO (2018). Long term effects of manual lymphatic drainage and active exercises on physical morbidities, lymphoscintigraphy parameters and lymphedema formation in patients operated due to breast cancer: a clinical trial. PloS one.

[15] Zielinski J, Jaworski R, Irga N, Kruszewski JW, Jaskiewicz J (2013). Analysis of selected factors influencing seroma formation in breast cancer patients undergoing mastectomy. Arch Med Sci AMS.

[16] Akinci M, Cetin B, Aslan S, Kulacoglu H (2009). Factors affecting seroma formation after mastectomy with full axillary dissection. Acta Chir Belg.

[17] Barwell J, Campbell L, Watkins RM, Teasdale C (1997). How long should suction drains stay in after breast surgery with axillary dissection?. Ann R Coll Surg Engl.

[18] Stanczyk M, Grala B, Zwierowicz T, Maruszynski M (2007). Surgical resection for persistent seroma, following modified radical mastectomy. World J Surg Oncol.

[19] Barton A, Blitz M, Callahan D, Yakimets W, Adams D, Dabbs K (2006). Early removal of postmastectomy drains is not beneficial: results from a halted randomized controlled trial. Am J Surg.

[20] Ulusoy AN, Polat C, Alvur M, Kandemir B, Bulut F (2003). Effect of fibrin glue on lymphatic drainage and on drain removal time after modified radical mastectomy: a prospective randomized study. Breast J.

[21] Vasileiadou K, Kosmidis C, Anthimidis G, Miliaras S, Kostopoulos I, Fahantidis E (2017). Cyanoacrylate adhesive reduces seroma production after modified radical mastectomy or quadrantectomy with lymph node dissection-a prospective randomized clinical trial. Clin Breast Cancer.

[22] Gupta R, Pate K, Varshney S, Goddard J, Royle GT (2001). A comparison of 5-day and 8-day drainage following mastectomy and axillary clearance. Eur J Surg Oncol.

[23] Cameron AE, Ebbs SR, Wylie F, Baum M (1988). Suction drainage of the axilla: a prospective randomized trial. Br J Surg.

[24] Saratzis A, Soumian S, Willetts R, Rastall S, Stonelake PS (2009). Use of multiple drains after mastectomy is associated with more patient discomfort and longer postoperative stay. Clin Breast Cancer.

[25] Kumar S, Lal B, Misra MC (1995). Post-mastectomy seroma: a new look into the aetiology of an old problem. J R Coll Surg Edinb.

[26] van Bemmel AJ, van de Velde CJ, Schmitz RF, Liefers GJ (2011). Prevention of seroma formation after axillary dissection in breast cancer: a systematic review. Eur J Surg Oncol.

[27] Clegg-Lamptey JN, Dakubo JC, Hodasi WM (2007). Comparison of four-day and ten-day post-mastectomy passive drainage in Accra, Ghana. East Afr Med J.

[28] Thomson DR, Sadideen H, Furniss D (2013). Wound drainage after axillary dissection for carcinoma of the breast. Cochrane Database Syst Rev.

[29] He XD, Guo ZH, Tian JH, Yang KH, Xie XD (2011). Whether drainage should be used after surgery for breast cancer? A systematic review of randomized controlled trials. Med Oncol.

[30] van Bastelaar J, Granzier R, van Roozendaal LM, Beets G, Dirksen CD, Vissers Y (2018). A multi-center, double blind randomized controlled trial evaluating flap fixation after mastectomy using sutures or tissue glue versus conventional closure: protocol for the Seroma reduction After Mastectomy (SAM) trial. BMC Cancer.

[31] de Rooij L, van Kuijk SMJ, van Haaren ERM, Janssen A, Vissers YLJ, Beets GL, van Bastelaar J (2020). A single-center, randomized, non-inferiority study evaluating seroma formation after mastectomy combined with flap fixation with or without suction drainage: protocol for the Seroma reduction and drAin fRee mAstectomy (SARA) trial. BMC Cancer.

